# Gait Generation and Its Energy Efficiency Based on Rat Neuromusculoskeletal Model

**DOI:** 10.3389/fnins.2019.01337

**Published:** 2020-01-17

**Authors:** Misaki Toeda, Shinya Aoi, Soichiro Fujiki, Tetsuro Funato, Kazuo Tsuchiya, Dai Yanagihara

**Affiliations:** ^1^Department of Life Sciences, Graduate School of Arts and Sciences, The University of Tokyo, Tokyo, Japan; ^2^Department of Aeronautics and Astronautics, Graduate School of Engineering, Kyoto University, Kyoto, Japan; ^3^Department of Physiology and Biological Information, School of Medicine, Dokkyo Medical University, Tochigi, Japan; ^4^Department of Mechanical Engineering and Intelligent Systems, Graduate School of Informatics and Engineering, The University of Electro-Communications, Tokyo, Japan

**Keywords:** rat, walk, trot, energy efficiency, central pattern generator, muscle synergy, neuromusculoskeletal model

## Abstract

Changing gait is crucial for adaptive and smooth animal locomotion. Although it remains unclear what makes animals decide on a specific gait, energy efficiency is an important factor. It has been reported that the relationship of oxygen consumption with speed is U-shaped for each horse gait and that different gaits have different speeds at which oxygen consumption is minimized. This allows the horse to produce energy-efficient locomotion in a wide speed range by changing gait. However, the underlying mechanisms causing oxygen consumption to be U-shaped and the speeds for the minimum consumption to be different between different gaits are unclear. In the present study, we used a neuromusculoskeletal model of the rat to examine the mechanism from a dynamic viewpoint. Specifically, we constructed the musculoskeletal part of the model based on empirical anatomical data on rats and the motor control model based on the physiological concepts of the spinal central pattern generator and muscle synergy. We also incorporated the posture and speed regulation models at the levels of the brainstem and cerebellum. Our model achieved walking through forward dynamic simulation, and the simulated joint kinematics and muscle activities were compared with animal data. Our model also achieved trotting by changing only the phase difference of the muscle-synergy-based motor commands between the forelimb and hindlimb. Furthermore, the speed of each gait varied by changing only the extension phase duration and amplitude of the muscle synergy-based motor commands and the reference values for the regulation models. The relationship between cost of transport (CoT) and speed was U-shaped for both the generated walking and trotting, and the speeds for the minimum CoT were different for the two gaits, as observed in the oxygen consumption of horses. We found that the resonance property and the posture and speed regulations contributed to the CoT shape and difference in speeds for the minimum CoT. We further discussed the energy efficiency of gait based on the simulation results.

## 1. Introduction

**Figure 1:**
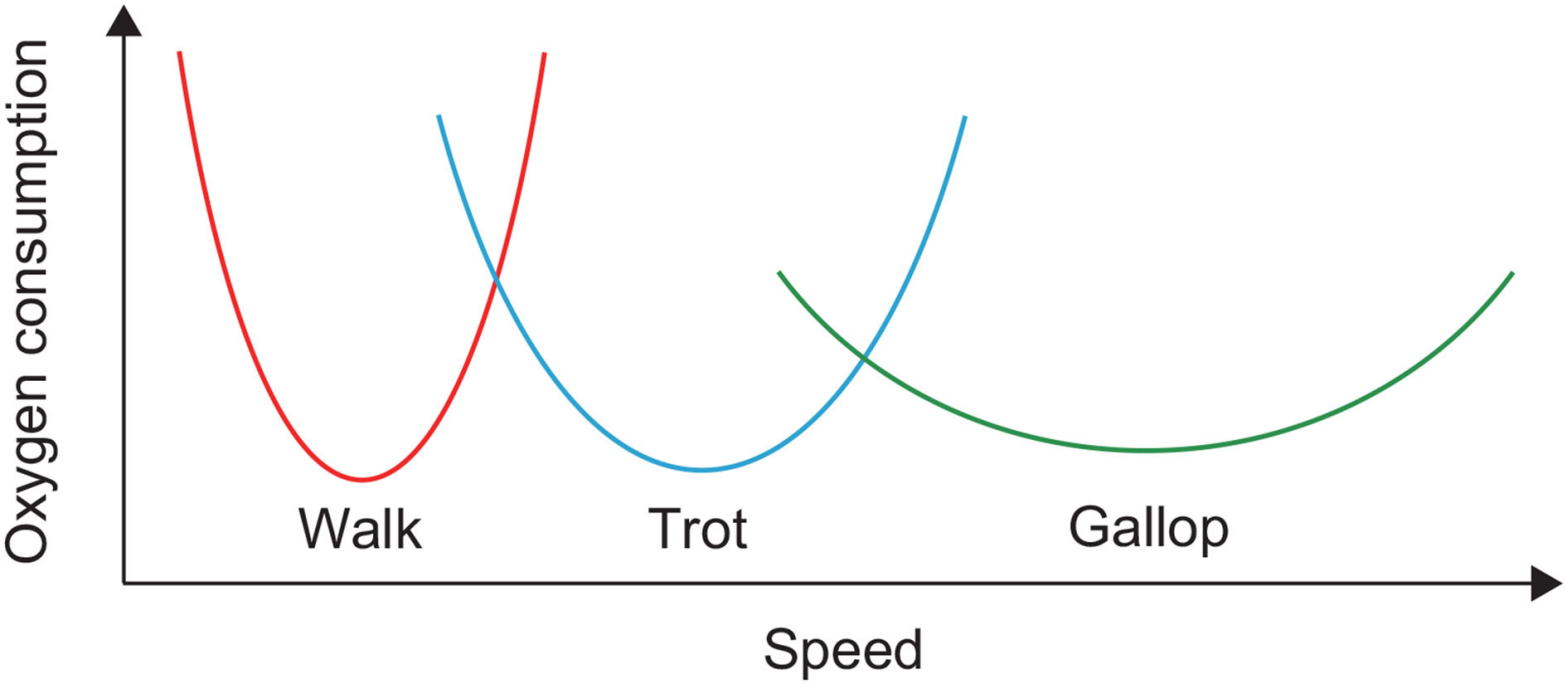
Oxygen consumption of horses in walking, trotting, and galloping.

Animals can generate adaptive and smooth locomotion in various conditions. One important strategy for such locomotion is the use of different gaits. For example, quadruped animals walk, amble, trot, pace, canter, and gallop. Although gait is the motor outcome of a complicated and redundant musculoskeletal system controlled by the central nervous system, it is largely unclear what makes animals decide on a gait. One important factor for deciding gait is the energy efficiency of locomotion; that is, animals want to minimize the cost of transport (CoT). In particular, it has been reported that the relation between oxygen consumption and speed is U-shaped for each horse gait and that different gaits have different speeds at which oxygen consumption is minimized (Figure 1) (Hoyt and Taylor, 1981). Walking, trotting, and galloping are energy-efficient at low, middle, and high speeds, respectively. Walking and trotting share a common speed range, as do trotting and galloping. Therefore, horses can produce energy-efficient locomotion over a wide speed range by changing their gait. However, the underlying mechanisms making the oxygen consumption U-shaped and the speeds for minimum consumption different between gaits remain unclear.

Locomotion is generated through interactions between the central nervous system, musculoskeletal system, and environment. It is difficult to fully analyze the locomotor mechanism with animal data alone. Recently, modeling studies have attracted attention because physiological findings and hypotheses can be used to develop reasonably realistic motor control models, and biomechanical and anatomical findings can be used to construct detailed musculoskeletal models (Ivashko et al., 2003; Yakovenko et al., 2004; Ekeberg and Pearson, 2005; Nishii, 2006; Aoi et al., 2013a; Fukuoka et al., 2015; Hunt et al., 2015; Aoi and Funato, 2016; Markin et al., 2016; Fujiki et al., 2018). Motor control and musculoskeletal models are integrated to produce locomotion through forward dynamics simulation. This allows the locomotor mechanism to be examined from a dynamic viewpoint.

In this study, we investigated the energy efficiency of gait using a rat neuromusculoskeletal model. Specifically, we constructed a musculoskeletal model composed of the trunk, forelimbs, and hindlimbs based on anatomical data. This model is an improvement on our previous rat hindlimb model (Aoi et al., 2013a). We also improved our previous motor control model to control the rat four-limb model. The motor control model was developed based on the hypothetical two-layer central pattern generator (CPG) model at the spinal cord level (Burke et al., 2001; Rybak et al., 2006) and the muscle synergy hypothesis (Tresch et al., 1999; Todorov and Jordan, 2002; d'Avella et al., 2003; Ting and Macpherson, 2005; Ivanenko et al., 2006; Drew et al., 2008; Takei et al., 2017), which describes a simple control strategy for redundant motor systems. Furthermore, we incorporated movement regulation models at the levels of the brainstem and cerebellum through brainstem descending pathways. We simulated the walking of our model and compared the simulation results with animal data. In addition, we simulated trotting and changed the speed of each gait using simple motor control strategies. We calculated the CoT of walking and trotting for the generated speeds and, in this paper, we discuss the energy efficiency of gait based on the simulation results.

## 2. Method

### 2.1. Musculoskeletal Model

**Figure 2:**
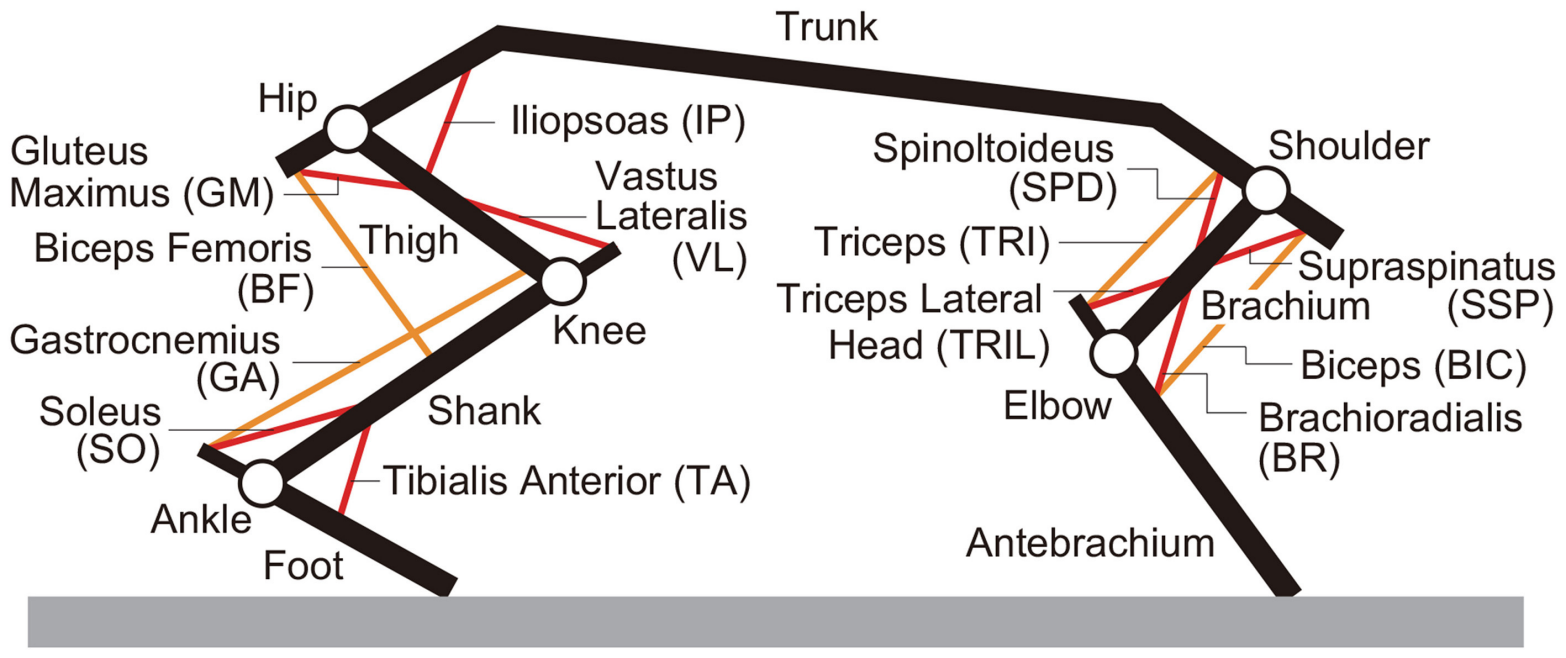
Musculoskeletal model.

We developed a rat musculoskeletal model based on our previous model, which focused on the hindlimbs without incorporating the forelimbs (Aoi et al., 2013a). The skeletal part of the model consists of eleven rigid links representing the trunk (including the head), forelimbs (two links), and hindlimbs (three links), as shown in Figure 2. This model is two-dimensional, and the walking behavior is constrained to the sagittal plane. When the brachium and antebrachium are in a straight line and perpendicular to the trunk, the shoulder angle is 120° and the elbow angle is 180°. When the thigh, shank, and foot are in a straight line and perpendicular to the trunk, the hip angle is 120° and the knee and ankle angles are both 180°. The joint angles increase as the joints extend. We modeled the contact between the limb tips and the ground using viscoelastic elements. We derived the equations of motion using Lagrangian equations and solved them using the fourth-order Runge-Kutta method with a time step of 0.02 ms.

For the muscle part of the model, we used six principal muscles for each forelimb: four uniarticular, namely shoulder extension (supraspinatus, SSP), shoulder flexion (spinoltoideus, SPD), elbow flexion (brachioradialis, BR), and elbow extension (triceps lateral head, TRIL), and two biarticular, namely shoulder extension and elbow flexion (biceps, BIC) and shoulder flexion and elbow extension (triceps, TRI), as shown in Figure 2. We used seven principal muscles for each hindlimb: five uniarticular, namely hip flexion (iliopsoas, IP), hip extension (gluteus maximus, GM), knee extension (vastus lateralis, VL), ankle flexion (tibialis anterior, TA), and ankle extension (soleus, SO), and two biarticular, namely hip extension and knee flexion (biceps femoris, BF) and knee flexion and ankle extension (gastrocnemius, GA). The moment arms of the muscles around the joints are constant, regardless of joint angles. Each muscle generates muscle tension *F*_*m*_ (m = SSP, SPD, BR, TRIL, BIC, TRI, IP, GM, VL, TA, SO, BF, and GA) through contractile and passive elements, which is given based on Aoi et al. (2013a) by

(1) $$\begin{array}{l} F_{m}=F_{m}^{\text{max}}\left(a_{m}F_{m}^{\text{l}}F_{m}^{\text{v}}+F_{m}^{\text{p}}\right) \end{array}$$

where $F_{m}^{\text{max}}$ is the maximum muscle tension, *a*_*m*_ is the muscle activation (0 ≤ *a*_*m*_ ≤ 1), $F_{m}^{\text{l}}$ is the force-length relationship, $F_{m}^{\text{v}}$ is the force-velocity relationship, and $F_{m}^{\text{p}}$ is the passive component. The muscle lengths were normalized by $l_{m}^{\text{max}}$, which was set so that all uniarticular muscles had a length of 85% of $l_{m}^{\text{max}}$ and all biarticular muscles had a length of 75% of $l_{m}^{\text{max}}$ at a neutral posture with the shoulder at 60°, the elbow at 85°, the hip at 70°, the knee at 90°, and the ankle at 100°. In addition, 2° of joint motion corresponded to 1% of muscle length change, except for BIC and GA (4.5° at the shoulder for BIC, 1.5° at the ankle or 4.5° at the knee for GA). The muscle contractile velocities were normalized by $1.8l_{m}^{\text{max}}$.

The muscle activation *a*_*m*_ is determined through

(2) $$\begin{array}{l} \tau_{\text{act}}{\dot{a}}_{m}+\left\{\frac{\tau_{\text{act}}}{\tau_{\text{deact}}}+\left(1-\frac{\tau_{\text{act}}}{\tau_{\text{deact}}}\right)u_{m}\right\}a_{m}=u_{m} \end{array}$$

where τ_act_ and τ_deact_ are respectively, activation and deactivation time constants (11 and 18 ms, respectively) and *u*_*m*_ is the motor command determined in the motor control model.

### 2.2. Motor Control Model

We developed a motor control model based on our previous work (Aoi et al., 2013a). It consists of the following two components: 1. a movement generator, which produces motor commands in a feedforward fashion at the spinal cord level to create periodic limb movements based on the muscle synergy hypothesis and 2. a movement regulator, which creates motor commands to regulate locomotor behavior in a feedback fashion at the brainstem and cerebellar levels based on proprioceptive and somatosensory information. The motor command *u*_*m*_ is the summation of the two components from the movement generator and the movement regulator, namely $u_{m}^{\text{Syn}}$ and $u_{m}^{\text{Reg}}$, respectively.

(3) $$\begin{array}{l} u_{m}=u_{m}^{\text{Syn}}+u_{m}^{\text{Reg}} \end{array}$$

#### 2.2.1. Movement Generator

The movement generator is based on the hypothetical two-layer CPG model composed of a rhythm generator (RG) network, which produces rhythm and phase information for motor commands, and a pattern formation (PF) network, which produces spatiotemporal patterns of motor commands (Burke et al., 2001; Rybak et al., 2006).

For the RG model, we used four simple phase oscillators, each of which produces a basic rhythm and phase information for the corresponding limb. We used $\phi_{i}^{j}$ (*i* = left, right, *j* = fore, hind) for the oscillator phase ($0\leq \phi_{i}^{j}<2\pi$), which follows the dynamics given by

(4) $$\begin{array}{l} {\dot{\phi }}_{\text{left}}^{\text{fore}}=\frac{2\pi }{T}-K_{1}\text{ sin}\left(\phi_{\text{left}}^{\text{fore}}-\phi_{\text{right}}^{\text{fore}}-\pi \right)-K_{2}\text{ sin}\left(\phi_{\text{left}}^{\text{fore}}-\phi_{\text{left}}^{\text{hind}}+\Delta \right)\\ {\dot{\phi }}_{\text{right}}^{\text{fore}}=\frac{2\pi }{T}-K_{1}\text{ sin}\left(\phi_{\text{right}}^{\text{fore}}-\phi_{\text{left}}^{\text{fore}}-\pi \right)-K_{2}\text{ sin}\left(\phi_{\text{right}}^{\text{fore}}-\phi_{\text{right}}^{\text{hind}}+\Delta \right)\\ {\dot{\phi }}_{\text{left}}^{\text{hind}}=\frac{2\pi }{T}-K_{1}\text{ sin}\left(\phi_{\text{left}}^{\text{hind}}-\phi_{\text{right}}^{\text{hind}}-\pi \right)-K_{2}\text{ sin}\left(\phi_{\text{left}}^{\text{hind}}-\phi_{\text{left}}^{\text{fore}}-\Delta \right)\\ {\dot{\phi }}_{\text{right}}^{\text{hind}}=\frac{2\pi }{T}-K_{1}\text{ sin}\left(\phi_{\text{right}}^{\text{hind}}-\phi_{\text{left}}^{\text{hind}}-\pi \right)-K_{2}\text{ sin}\left(\phi_{\text{right}}^{\text{hind}}-\phi_{\text{right}}^{\text{fore}}-\Delta \right) \end{array}$$

where *T* is the gait cycle duration and *K*_1_ and *K*_2_ are gain parameters. The second term on the right-hand side ensures that the left and right limbs move in antiphase to maintain interlimb coordination. The third term on the right-hand side ensures that the ipsilateral limbs move in relative phase of Δ to maintain interlimb coordination.

**Figure 3:**
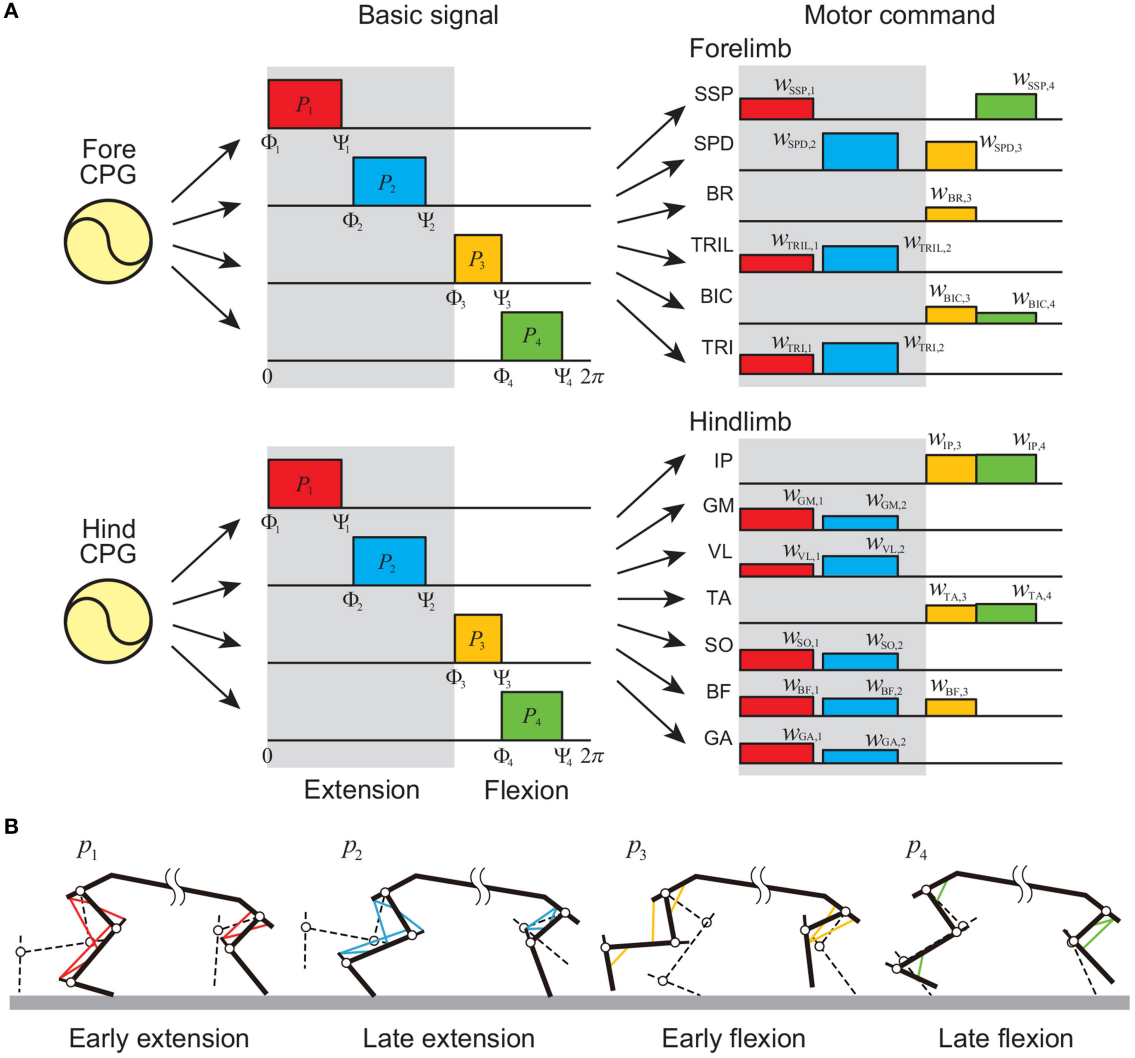
Movement generator. **(A)** Motor commands as linear combinations of four rectangular pulses in each forelimb and hindlimb. Gray regions indicate extension phases, and others indicate flexion phases. **(B)** Activated muscles at each pulse.

For the PF model, we determined the motor commands necessary to produce periodic limb movements in accordance with the corresponding oscillator phase based on the muscle synergy hypothesis, which suggests that the linear combination of only a small number of basic signals produces a large portion of motor commands in animal locomotion (Ivanenko et al., 2006; Dominici et al., 2011; Markin et al., 2012; Catavitello et al., 2015; Rigosa et al., 2015). Specifically, we used four rectangular pulses *p*_*i*_ (*i* = 1, …, 4) for each limb, which are given by

(5) $$\begin{array}{l} p_{i}\left(\phi \right)=\left\{ \begin{array}{cc} 1 & \Phi_{i}\leq \phi <{\text{Ψ}}_{i}\\ 0 & \text{otherwise} \end{array}\;i=1,\ldots ,4\right. \end{array}$$

where Φ_*i*_ and Ψ_*i*_ (*i* = 1, …, 4) are the onset and end phases of the pulse, respectively, and we omitted the suffix of ϕ. *p*_1_, *p*_2_, *p*_3_, and *p*_4_ contribute to early extension, late extension, early flexion, and late flexion, respectively, as shown in Figure 3 [extension and flexion phases start at Φ_1_ (= 0 rad) and Φ_3_, respectively]. We used the same values of Φ_*i*_ and Ψ_*i*_ for the four limbs irrespective of whether they were forelimb or hindlimb. The motor command $u_{m}^{\text{Syn}}$ of the movement generator is given by

(6) $$\begin{array}{l} u_{m}^{\text{Syn}}=\overset{4}{\underset{i=1}{\sum }}w_{m,i}p_{i}\left(\phi \right) \end{array}$$

where *w*_*m, i*_ (*i* = 1, …, 4) is the weighting coefficient.

#### 2.2.2. Movement Regulator

**Figure 4:**
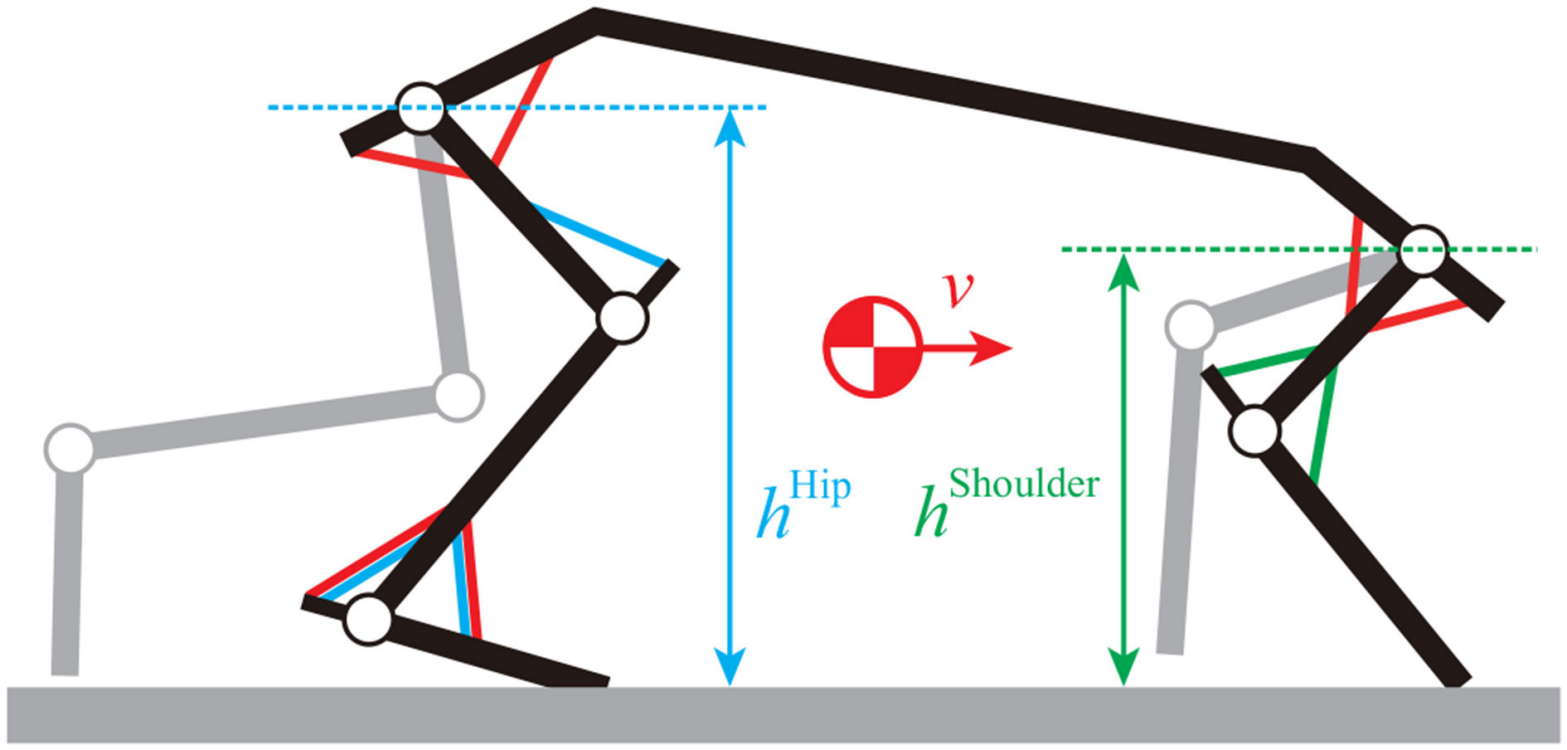
Movement regulator based on shoulder height, hip height, and forward speed. BR and TRIL muscles are used for shoulder height, VL, TA, and SO muscles are used for hip height, and SSP, SPD, IP, GM, TA, and SO muscles are used for speed.

At the levels of the brainstem and cerebellum, locomotor behavior is regulated based on proprioceptive and somatosensory information (Takakusaki, 2017). For the rat, it is crucial to maintain body height and forward speed during locomotion (Figure 4). For simplicity, we focused on these two factors.

For the body height, we used simple feedback control for the standing limb. For the forelimbs, we used the BR and TRIL muscles to maintain the shoulder height. The motor command $p_{m}^{\text{height}}$ (*m* = BR and TRIL) is given by

(7) $$\begin{array}{l} p_{m}^{\text{height}}\\ =\left\{ \begin{array}{cc} -K_{m}^{\text{height}}\left(h^{\text{Shoulder}}-h_{0}^{\text{Shoulder}}\right)-D_{m}^{\text{height}}{\dot{h}}^{\text{Shoulder}} & \text{in stance phase}\\ 0 & \text{otherwise} \end{array}\right. \end{array}$$

where *h*^Shoulder^ and ${\dot{h}}^{\text{Shoulder}}$ are the shoulder height and its rate, respectively, $h_{0}^{\text{Shoulder}}$ is the reference height, and $K_{m}^{\text{height}}$ and $D_{m}^{\text{height}}$ are gain parameters. For the hindlimbs, we used the VL, TA, and SO muscles to maintain the hip height. The motor command $p_{m}^{\text{height}}$ (*m* = VL, TA, and SO) is given by

(8) $$\begin{array}{l} p_{m}^{\text{height}}=\left\{ \begin{array}{cc} -K_{m}^{\text{height}}\left(h^{\text{Hip}}-h_{0}^{\text{Hip}}\right)-D_{m}^{\text{height}}{\dot{h}}^{\text{Hip}} & \text{in stance phase}\\ 0 & \text{otherwise} \end{array}\right. \end{array}$$

where *h*^Hip^ and ${\dot{h}}^{\text{Hip}}$ are the hip height and its rate, respectively, and $h_{0}^{\text{Hip}}$ is the reference height.

For the forward speed, we used simple feedback control for the standing limb. We used the SSP, SPD, IP, GM, TA, and SO muscles to maintain speed. The motor command $p_{m}^{\text{speed}}$ (*m* = SSP, SPD, IP, GM, TA, and SO) is given by

(9) $$\begin{array}{l} p_{m}^{\text{speed}}=\left\{ \begin{array}{cc} -K_{m}^{\text{speed}}\left(v-v_{0}\right) & \text{in stance phase}\\ 0 & \text{otherwise} \end{array}\right. \end{array}$$

where *v* is the forward speed, *v*_0_ is its desired value, and $K_{m}^{\text{speed}}$ is a gain parameter.

The summation of these elements produces the motor command of the movement regulator. Because regulation is managed at the brainstem and cerebellar levels, the command signals are delayed and the motor command $u_{m}^{\text{Reg}}$ of the movement regulator is given by

(10) $$\begin{array}{l} u_{m}^{\text{Reg}}\left(t\right)=u_{m}^{\text{height}}\left(t\right)+u_{m}^{\text{speed}}\left(t\right) \end{array}$$

where

(11) $$\begin{array}{lll} u_{m}^{\text{height}}\left(t\right) & = & p_{m}^{\text{height}}\left(t-\tau^{\text{Delay}}\right)\\ u_{m}^{\text{speed}}\left(t\right) & = & p_{m}^{\text{speed}}\left(t-\tau^{\text{Delay}}\right) \end{array}$$

and τ^Delay^ (= 15 ms) is the delay between receiving the transmission of proprioceptive and somatosensory information at the brainstem and cerebellar levels and sending the motor command to the spinal cord level.

### 2.3. Changing Gait and Speed

**Figure 5:**
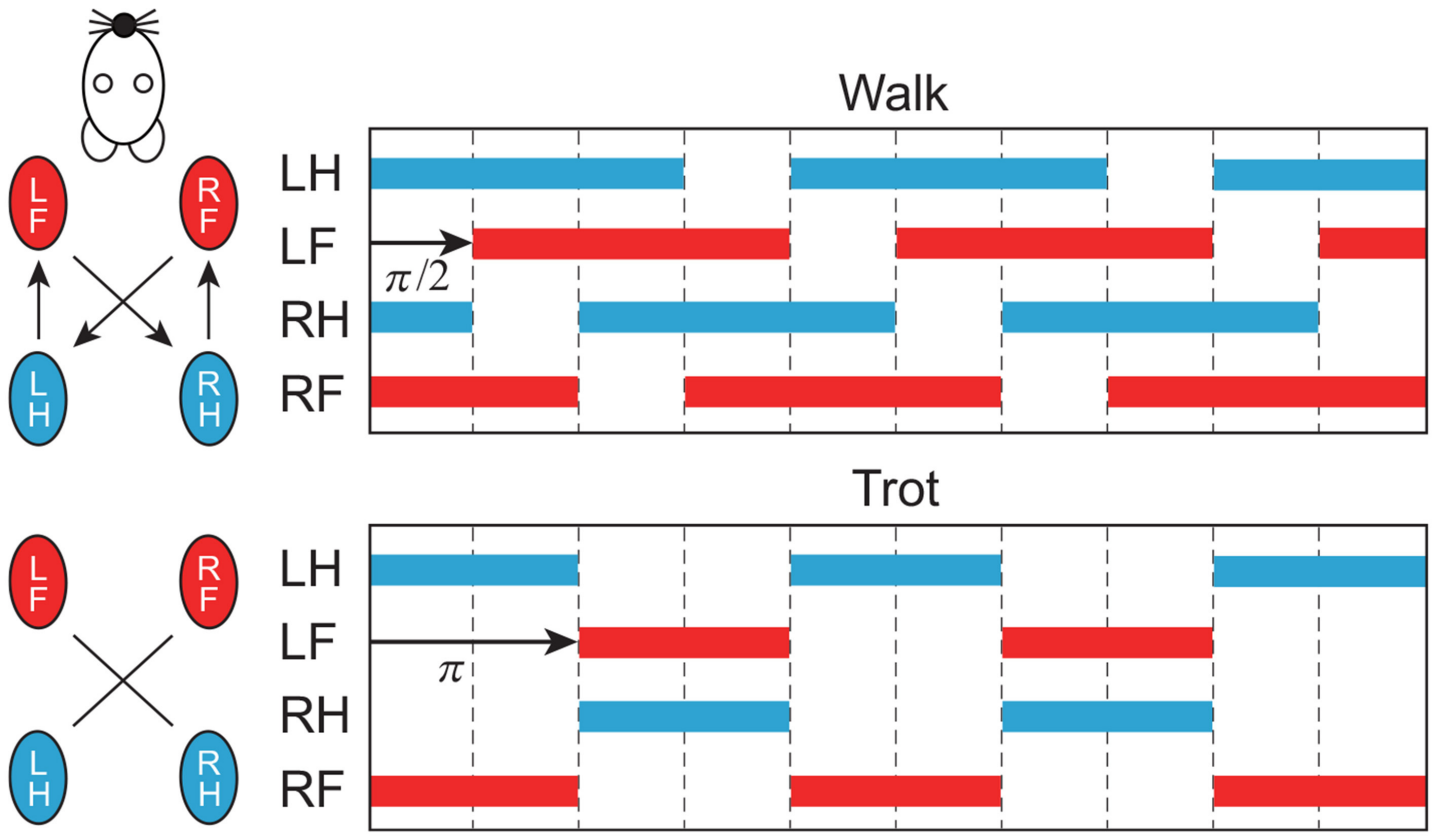
Schematic diagram of footfall sequence for walking and trotting. Color bars indicate stance phase. Right and left limbs move in antiphase. Relative phase between forelimb and hindlimb is π/2 for walking and π for trotting (LF, left fore; LH, left hind; RF, right fore; RH, right hind).

In this study, we focused on two gaits, namely walking and trotting. They are mainly classified by the footfall sequence. Specifically, four limbs move out of phase in walking, and diagonal limbs are paired in trotting (Figure 5). Right and left limbs move in antiphase in both walking and trotting. The major difference between the gaits is the relative phase between the ipsilateral limbs. To change the relative phase of the limb movements, we changed the relative phase of the muscle-synergy-based motor command $u_{m}^{\text{Syn}}$ between the ipsilateral limbs by changing Δ in (4). In particular, we used Δ = π/2 for walking and Δ = π for trotting.

**Figure 6:**
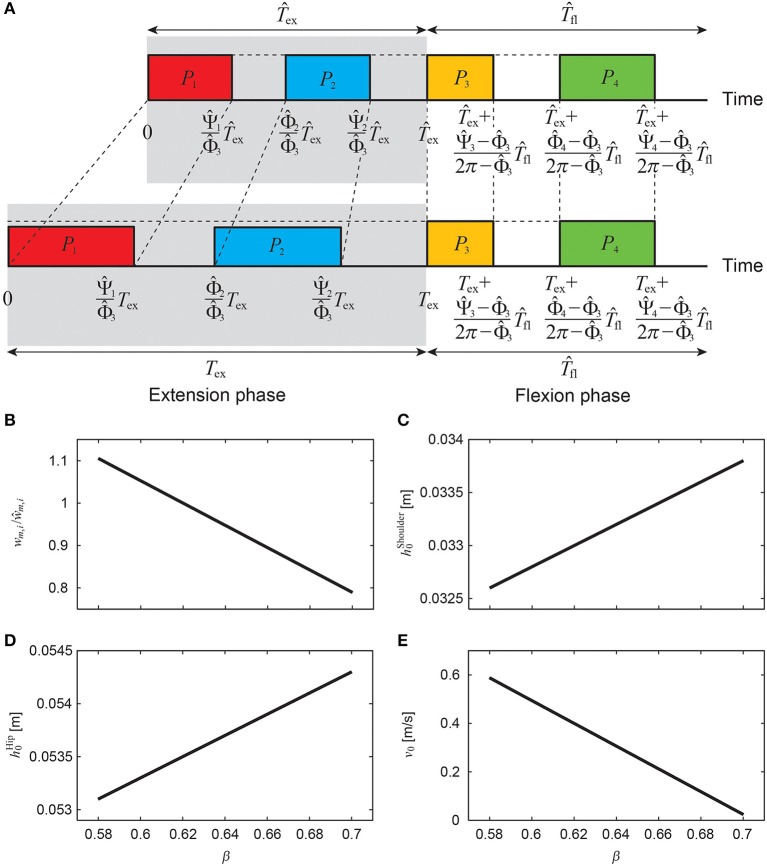
Regulation of muscle-synergy-based motor command **(A)** to change speed by changing extension phase duration *T*_ex_ (= β*T*) while keeping flexion phase duration *T*_fl_ unchanged, where weighting coefficient *w*_*m, i*_/*ŵ*_*m, i*_ **(B)**, reference shoulder height $h_{0}^{\text{Shoulder}}$ **(C)**, reference hip height $h_{0}^{\text{Hip}}$ **(D)**, and reference speed *v*_0_ **(E)** depend on β.

Animals change the gait cycle duration to vary speed, where the duration of the flexion phase for swinging the limb remains almost unchanged and the duration of the extension phase for supporting the body and producing the propulsive forces is changed substantially (Goslow et al., 1973; Heglund and Taylor, 1998; Clarke and Still, 1999; Górska et al., 1999; Yakovenko et al., 2005). In this study, we changed the speed by changing the duration of the extension phase *T*_ex_ (= β*T*) using β while keeping the duration of the flexion phase *T*_fl_ unchanged (*T* = *T*_ex_ + *T*_fl_ = *T*_fl_/(1 − β)), as shown in Figure 6A. For the nominal speed, which we determined from animal data as explained below, we used $\hat{\beta }$, ${\hat{T}}_{\text{ex}}$, ${\hat{\Phi }}_{i}$, ${\hat{\text{Ψ}}}_{i}$, *ŵ*_*m, i*_ (*i* = 1, …, 4), ${\hat{h}}_{0}^{\text{Shoulder}}$, ${\hat{h}}_{0}^{\text{Hip}}$, and ${\hat{v}}_{0}$ for motor control parameters β, *T*_ex_, Φ_*i*_, Ψ_*i*_, *w*_*m, i*_ (*i* = 1, …, 4), $h_{0}^{\text{Shoulder}}$, $h_{0}^{\text{Hip}}$, and *v*_0_, respectively. The onset phase Φ_*i*_ and end phase Ψ_*i*_ (*i* = 1, …, 4) of each pulse are given by

(12) $$\begin{array}{lll} \Phi_{i} & = & \left\{ \begin{array}{cc} \frac{\beta }{\hat{\beta }}{\hat{\Phi }}_{i} & i=1,2\\ \frac{1-\beta }{1-\hat{\beta }}{\hat{\Phi }}_{i}+\frac{2\pi \left(\beta -\hat{\beta }\right)}{1-\hat{\beta }} & i=3,4 \end{array}\right.\\ {\text{Ψ}}_{i} & = & \left\{ \begin{array}{cc} \frac{\beta }{\hat{\beta }}{\hat{\text{Ψ}}}_{i} & i=1,2\\ \frac{1-\beta }{1-\hat{\beta }}{\hat{\text{Ψ}}}_{i}+\frac{2\pi \left(\beta -\hat{\beta }\right)}{1-\hat{\beta }} & i=3,4 \end{array}\right. \end{array}$$

We decreased (increased) the extension phase duration to increase (decrease) the speed, which decreased (increased) the duration of pulses of the extension phase. To prevent the model from decreasing (increasing) the speed, we increased (decreased) the weighting coefficients *w*_*m, i*_ (*i* = 1, 2) of the muscle-synergy-based rectangular pulses for the extension phase (Figure 6B) as

(13) $$\begin{array}{l} w_{m,i}=\frac{1-\beta }{1-\hat{\beta }}{\hat{w}}_{m,i}\;i=1,2 \end{array}$$

As we changed the locomotion speed, we also changed the reference height (shoulder, hip) and speed for the movement regulator (Figures 6C–E) as

(14) $$\begin{array}{lll} h_{0}^{\text{Shoulder}} & = & {\hat{h}}_{0}^{\text{Shoulder}}+\alpha^{\text{Shoulder}}\left(\beta -\hat{\beta }\right)\\ h_{0}^{\text{Hip}} & = & {\hat{h}}_{0}^{\text{Hip}}+\alpha^{\text{Hip}}\left(\beta -\hat{\beta }\right)\\ v_{0} & = & {\hat{v}}_{0}+\alpha^{\text{Speed}}\left(\beta -\hat{\beta }\right) \end{array}$$

where α^Shoulder^, α^Hip^, and α^Speed^ are coefficients.

### 2.4. Model Parameters

#### 2.4.1. Parameters for the Musculoskeletal Model

To determine the physical parameters of the musculoskeletal model, we used seven adult male Wistar rats (body weight: 125 ± 10 g). The rats were deeply anesthetized, and their musculoskeletal features were measured. The experiments were approved by the Ethical Committee for Animal Experiments at the University of Tokyo and carried out in accordance with the Guidelines for Research with Experimental Animals of the University of Tokyo and the Guide for the Care and Use of Laboratory Animals (NIH Guide).

For the skeletal model, we measured several physical parameters of the rats, such as masses, joint positions, and distances between joints, and determined the model parameters from these measurements, as shown in Table 1. For the muscle model, we first electrically stimulated individual muscles and determined which joint movements were needed to verify our musculoskeletal model. We measured the attachment, direction, and physiological cross-sectional area (PCSA) for each muscle and determined the model parameters from these measurements, as shown in Table 2, where the maximum muscle tension $F_{m}^{\text{max}}$ was determined based on the measured PCSA and the moment arms were determined from the center of the range of joint movement during locomotion.

**Table 1:** Physical parameters of skeletal model.

**Parameter**	**Trunk**	**Brachium**	**Antebrachium**	**Thigh**	**Shank**	**Foot**
Mass (g)	99.8	1.6	1.6	5.2	2.8	1.5
Length (mm)	93.0	16.1	19.0	18.5	27.2	17.7
MOI (×10^2^ gmm^2^)	1410	0.57	0.53	5.73	2.62	0.75

*MOI, moment of inertia around center of mass*.

**Table 2:** Physical parameters of muscle model.

**Parameter**	**SSP**	**SPD**	**BR**	**TRIL**	**BIC**	**TRI**	
$F_{m}^{\text{max}}$ (N)	11.1	9.8	5.1	11.5	7.7	23.2	
MA (mm)	3.6	5.3	3.6	5.9	2.4 (s)	5.7 (s)	
					4.0 (e)	5.9 (e)	
**Parameter**	**IP**	**GM**	**VL**	**TA**	**SO**	**BF**	**GA**
$F_{m}^{\text{max}}$ (N)	15.7	23.3	24.0	4.1	3.5	3.1	4.5
MA (mm)	4.5	2.3	3.2	5.1	6.0	2.5 (h)	4.2 (k)
						12.5 (k)	6.0 (a)

*MA, moment arm of muscle around joint; s, shoulder; e, elbow; h, hip; k, knee; and a, ankle*.

#### 2.4.2. Parameters for the Motor Control Model

Based on measured data for rats walking on a treadmill at a speed of 0.4 m/s (Aoi et al., 2013a), we set the durations of the flexion and extension phases for the nominal speed as *T*_fl_ = 0.10 s and ${\hat{T}}_{\text{ex}}=0.16$ s, respectively ($\hat{\beta }=0.62$). We determined the motor control parameters for the nominal speed as follows so that our model achieved steady walking based on our previous results of the hindlimb model (Aoi et al., 2013a): ${\hat{\Phi }}_{1}=0$, ${\hat{\Phi }}_{2}=0.40\pi$, ${\hat{\Phi }}_{3}=1.24\pi$, ${\hat{\Phi }}_{4}=1.42\pi$, ${\hat{\text{Ψ}}}_{1}=0.33\pi$, ${\hat{\text{Ψ}}}_{2}=0.89\pi$, ${\hat{\text{Ψ}}}_{i}=1.42\pi$, ${\hat{\text{Ψ}}}_{i}=1.71\pi$, *ŵ*_*SSP, 1*_ = 0.24, *ŵ*_*SSP, 4*_ = 0.20, *ŵ*_*SPD, 2*_ = 0.27, *ŵ*_*SPD, 3*_ = 0.08, *ŵ*_*BR, 3*_ = 0.09, *ŵ*_*TRIL, 1*_ = 0.47, *ŵ*_*TRIL, 2*_ = 0.57, *ŵ*_*BIC, 3*_ = 0.17, *ŵ*_*BIC, 4*_ = 0.08, *ŵ*_*TRI, 1*_ = 0.27, *ŵ*_*TRI, 2*_ = 0.56, *ŵ*_*IP, 3*_ = 0.32, *ŵ*_*IP, 4*_ = 0.32, *ŵ*_*GM, 1*_ = 0.61, *ŵ*_*GM, 2*_ = 0.25, *ŵ*_*VL, 1*_ = 0.19, *ŵ*_*VL, 2*_ = 0.22, *ŵ*_TA, 3_ = 0.45, *ŵ*_TA, 4_ = 0.06, *ŵ*_*SO, 1*_ = 0.58, *ŵ*_*SO, 2*_ = 0.14, *ŵ*_*BF, 1*_ = 0.22, *ŵ*_BF, 2_ = 0.12, *ŵ*_BF, 3_ = 0.09, *ŵ*_*GA, 1*_ = 0.47, *ŵ*_GA, 2_ = 0.10, *ŵ*_*m, i*_ = 0 for the other values of *m* and *i*, ${\hat{h}}_{0}^{\text{Shoulder}}=0.033$ m, ${\hat{h}}_{0}^{\text{Hip}}=0.054$ m, ${\hat{v}}_{0}=0.4$ m/s, $K_{\text{BR}}^{\text{height}}=-2.07$, $K_{\text{TRIL}}^{\text{height}}=2.07$, $K_{\text{VL}}^{\text{height}}=12.4$, $K_{\text{TA}}^{\text{height}}=-12.4$, $K_{\text{SO}}^{\text{height}}=12.4$, $D_{\text{BR}}^{\text{height}}=-0.001$, $D_{\text{TRIL}}^{\text{height}}=0.001$, $D_{\text{VL}}^{\text{height}}=0.006$, $D_{\text{TA}}^{\text{height}}=-0.006$, $D_{\text{SO}}^{\text{height}}=0.006$, $K_{\text{SSP}}^{\text{speed}}=-0.007$, $K_{\text{SPD}}^{\text{speed}}=0.007$, $K_{\text{IP}}^{\text{speed}}=-0.052$, $K_{\text{GM}}^{\text{speed}}=0.052$, $K_{\text{TA}}^{\text{speed}}=-0.026$, $K_{\text{SO}}^{\text{speed}}=0.026$, *K*_1_ = 20, and *K*_2_ = 10. In addition, we set the coefficients for the regulation of the references in the movement regulator to change the speed as α^Shoulder^ = 0.01 m, α^Hip^ = 0.01 m, and α^Speed^ = −4.7 m/s.

### 2.5. Comparison With Animal Data

To evaluate our neuromusculoskeletal model, we compared the simulation results for walking with animal data. We used the joint angles of the hindlimbs measured in Aoi et al. (2013a), where rats walked on a treadmill at a speed of 0.4 m/s, and the joint angles of the forelimbs measured in Aoki et al. (2013), where intact rats walked at the average speed of 0.36 m/s in a custom-made runway box (length: 140 cm; width: 14 cm).

We used the electromyographic (EMG) data measured from the muscles of the hindlimbs in Aoi et al. (2013a) and the EMG data measured from two muscles (BIC and TRI) of the forelimbs in Aoki et al. (2013). Because we could not find EMG data for four muscles (SSP, SPD, BR, and TRIL) of the forelimbs of rats, we used EMG data for these muscles in cats, whose gait and joint movements are similar to those of rats, given in Drew et al. (2008), where cats walked on a treadmill at a speed of 0.35–0.45 m/s. In the comparison with the simulation results, we showed the EMG data so that their magnitudes are similar to those of simulated muscle activities.

### 2.6. Evaluation of Cost of Transport

The energetic cost of locomotion for our simulation results for walking and trotting was estimated based on the mechanical energy exerted by muscles. Based on previous work (Ogihara et al., 2011), we calculated the CoT ε as

(15) $$\begin{array}{l} \varepsilon =\frac{W}{D} \end{array}$$

where

$$\begin{array}{l} W=\eta_{+}+\eta_{-}\\ \eta_{+}=\underset{T}{\int }\underset{m}{\sum }F_{m}{\left[v_{m}\right]}^{+}dt\\ \eta_{-}=\frac{1}{4}\underset{T}{\int }\underset{m}{\sum }F_{m}{\left[-v_{m}\right]}^{+}dt \end{array}$$

*v*_*m*_ is the contracting velocity of the muscle (positive for contraction), and [*x*]^+^ is *x* if *x* ≥ 0 and 0 if *x* < 0. η_+_ and η_−_ are the positive and negative mechanical work done by muscles, respectively, for one gait cycle duration. The negative mechanical work was divided by four based on Margaria et al. (1963); Elmer and LaStayo (2014). *D* is the moving distance of the model for one gait cycle duration, which corresponds to the stride length.

In this study, the motor command *u*_*m*_ is generated by three elements: rectangular pulses $u_{m}^{\text{Syn}}$ in the movement generator and motor commands $u_{m}^{\text{height}}$ and $u_{m}^{\text{speed}}$ to regulate the posture and speed, respectively, in the movement regulator ($u_{m}=u_{m}^{\text{Syn}}+u_{m}^{\text{height}}+u_{m}^{\text{speed}}$). Because they determined the muscle activation *a*_*m*_ in (2), we calculated $a_{m}^{\text{Syn}}$, $a_{m}^{\text{height}}$, and $a_{m}^{\text{speed}}$ from $u_{m}^{\text{Syn}}$, $u_{m}^{\text{height}}$, and $u_{m}^{\text{speed}}$, respectively. Using these values, we calculated the CoTs ε^Syn^, ε^height^, and ε^speed^ from the three elements to investigate their contributions.

## 3. Results

### 3.1. Simulation of Walking

**Figure 7:**
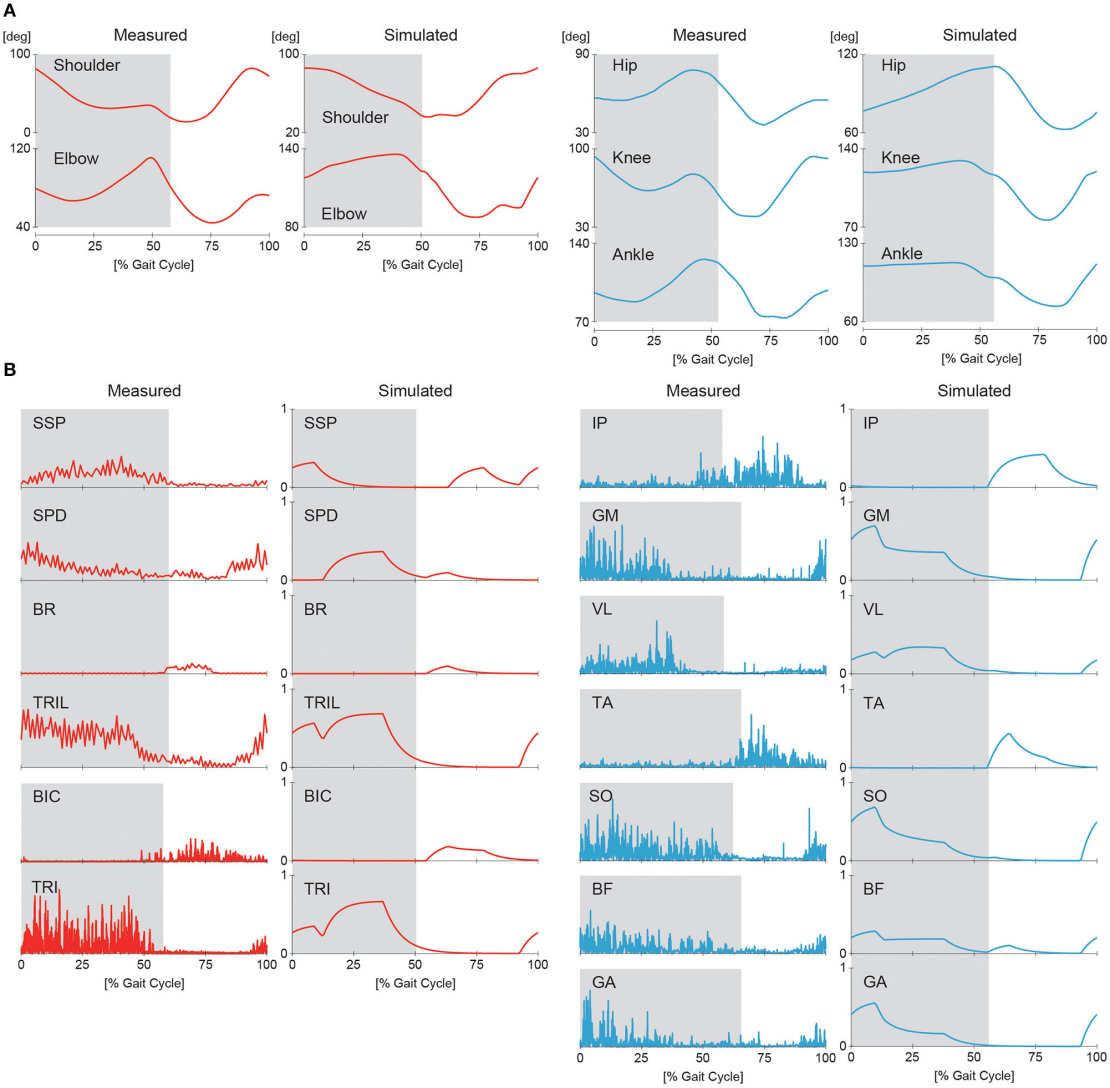
Simulated joint kinematics **(A)** and muscle activations **(B)** in walking compared with measured animal data. Gray regions indicate stance phases.

First, we conducted a computer simulation of our neuromusculoskeletal model for the nominal speed of walking using $\beta =\hat{\beta }$ and Δ = π/2 (see Supplementary Movie S1). The generated average speed was 0.2 m/s. Figures 7A,B show the joint angle and muscle activity, respectively, from the simulation compared with animal data. The simulation results show activity patterns similar to those of animals in terms of kinematics and muscle activity levels. However, our model was limited in its ability to accurately reproduce the locomotor behavior observed in animals. In particular, the elbow and knee joints were more extended than those of animals, which resulted in a shorter stride and slower speed than desired. The more extended elbow joint partly occurred because we did not incorporate the hand and wrist in the forelimb, and the forward speed was reduced by large ground reaction forces at the tips of the forelimbs. Similarly, the more extended knee joint partly occurred because we did not incorporate the phalangeal part in the hindlimb. The absence of flexibility of the spine in the trunk is another factor causing the extended posture. In addition, the activity of the SSP muscle appeared in a phase different from that of measured data. The SSP muscle in animals was activated in the same phase as that of the antagonistic SPD muscle so that the shoulder joint stiffness increased. In contrast, the SSP muscle in our model was activated in the same phase as that of the ipsilateral BR and BIC muscles.

### 3.2. Changing Gait and Speed

**Figure 8:**
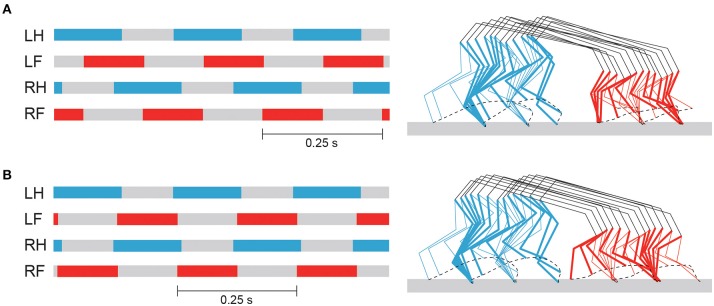
Footfall pattern and stick diagram of simulated walking **(A)** and trotting **(B)**. Stick diagram shows simulated locomotor behavior between two successive foot contacts of right hindlimb, where bold lines indicate right limbs. See Supplementary Movies S1, S2 for simulated walking and trotting, respectively.

By changing Δ from π/2 to π, our model achieved steady trotting (see Supplementary Movie S2). Although this gait had activity patterns almost identical to those of walking in terms of joint kinematics and muscle activations (Figure 7), the footfall pattern was different, as shown in Figure 8. The difference of the footfall pattern caused the difference in the trunk movement. In particular, while walking has a slight pitching movement of the trunk, trotting has almost no pitching movement, as shown in Figure 8 (see Supplementary Movies S1, S2).

**Figure 9:**
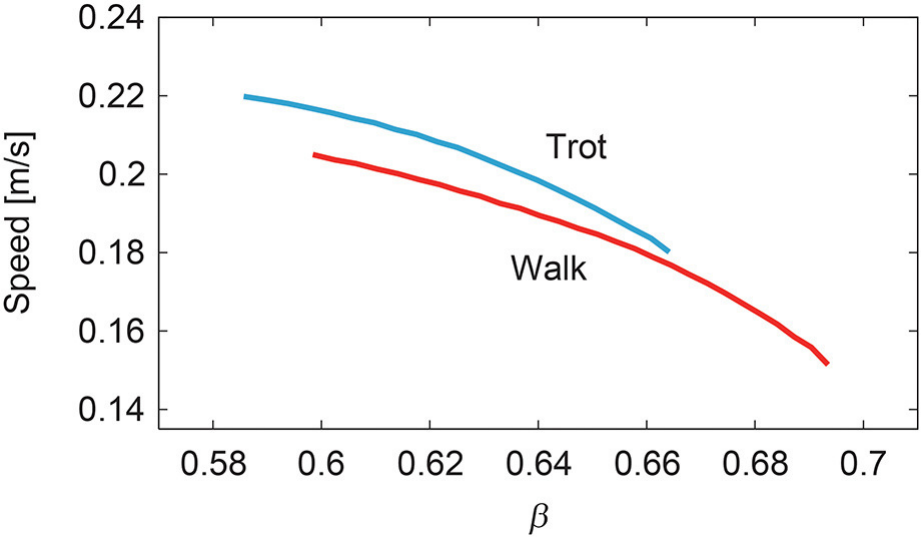
Generated speed during walking and trotting.

To change the speed of each gait, we slowly increased or decreased β from $\hat{\beta }$ while changing the duration of the extension phase, the amplitude of the muscle-synergy-based motor commands, and the reference values for the movement regulator based on β, as in (12–14). Figure 9 shows the speed of the simulated walking and trotting. Our model achieved speeds of 0.15–0.2 m/s for walking and 0.18–0.22 m/s for trotting. Trotting was faster than walking in each β.

### 3.3. Cost of Transport

**Figure 10:**
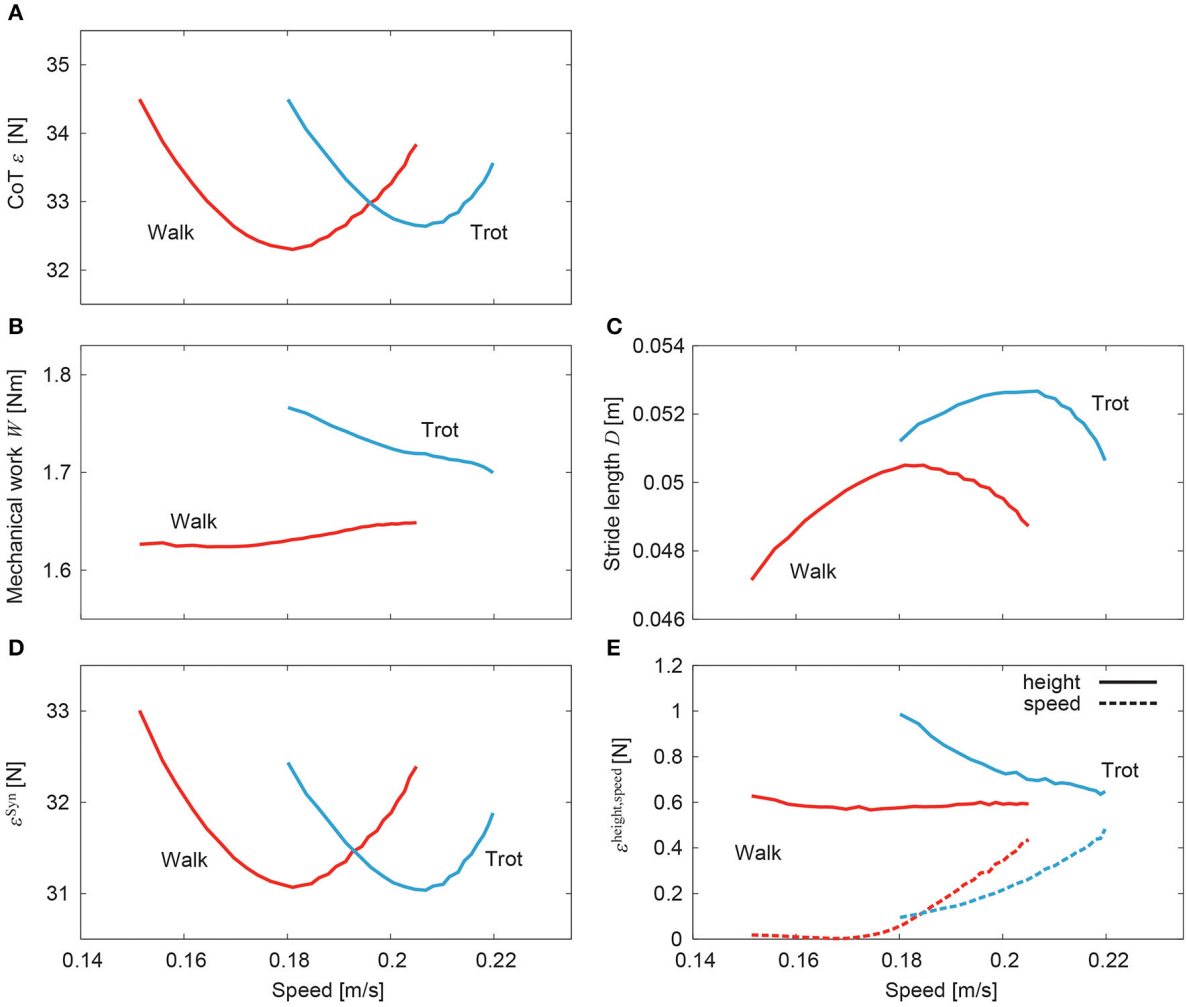
Cost of transport for speed of simulated walking and trotting. **(A)** Total CoT, **(B)** mechanical work, **(C)** stride length, **(D)** CoT contribution of muscle synergy-based pulses, and **(E)** CoT contributions of posture and speed regulators.

Figure 10A shows the CoT ε of walking and trotting for the generated speeds in the simulation. Both CoT curves are U-shaped. The speeds for the minimum CoT for walking and trotting are very different. Walking had lower (higher) CoTs than trotting at slow (fast) speed. The CoT was obtained by dividing the mechanical work *W* for one gait cycle duration by the stride length *D*, as in (15). Figures 10B,C show the mechanical work and stride length, respectively, with speed. The mechanical work slightly but monotonically increased in walking and decreased in trotting. In contrast, the stride length shows a single-peaked shape for speed in both walking and trotting. The speeds for the minimum CoT and maximum stride length were almost identical in both walking and trotting.

Figures 10D,E, respectively, show the contributions of the muscle synergy-based pulses and posture and speed regulators to the CoT (ε^Syn^, and ε^height^ and ε^speed^, respectively). The CoT contribution of the muscle synergy-based pulses was U-shaped in both walking and trotting and was the largest among the three elements. The CoT contributions of the posture and speed regulators were small. While the contribution of the posture regulator remained constant with speed in walking, it decreased in trotting. The contribution of the speed regulator increased in both walking and trotting.

## 4. Discussion

In this study, we improved our previous musculoskeletal model of rat hindlimbs (Aoi et al., 2013a) to construct a whole-body rat musculoskeletal model, which consists of the trunk, forelimbs, and hindlimbs. We also improved our motor control model (Aoi et al., 2013a) based on the muscle synergy hypothesis to control the whole-body rat model. Although the motor control model had a large number of motor control parameters, the rat model could be made to walk or trot by changing only the phase difference of the muscle-synergy-based motor commands between the forelimb and hindlimb (Figures 7, 8). Furthermore, the speed of each gait could be varied by changing only the duration of the extension phase, the amplitude of the muscle-synergy-based motor commands, and the reference values for the movement regulator (Figure 9). The relation between speed and CoT was U-shaped for both the walking and trotting generated, and the speeds for the minimum CoT were different for the two gaits, as observed in the oxygen consumption of animals (Figure 10).

### 4.1. Characteristics of Cost of Transport

For our simulation, the CoT vs. speed curves were U-shaped for both walking and trotting (Figure 10A). Walking had lower (higher) CoTs than trotting at slow (fast) speed. The CoTs were the same at a certain middle speed. These results indicate that walking and trotting are energy-efficient at slow and fast speeds, respectively. These trends are similar to those observed for animals (Figure 1).

The CoT was calculated by dividing the mechanical work of one gait cycle duration by the stride length, as shown in (15). The stride length showed a single-peaked shape against speed (Figure 10C). The speeds for the minimum CoT and maximum stride length were almost identical. We decreased the extension phase duration to increase the speed, which decreased the gait cycle duration. Because we increased the muscle-synergy-based motor commands during the extension phase as in (13), the stride length increased. However, this increase of the stride length was limited due to the increase of the gait frequency (decrease of the gait cycle duration). The stride length decreased over a critical frequency, which suggests a resonance property of the musculoskeletal dynamics and motor control input. Although these trends were similar between walking and trotting, the maximum stride length differed. These characteristics contributed majorly to the different energy efficiencies of gait. It has been reported that when the locomotor frequency increased, the stretch receptor of the hip prevented the hindlimbs from extending further (Mayer et al., 2018; Santuz et al., 2019). In the future, we would like to incorporate this sensory regulation model to control the stride length to investigate the mechanism of energy-efficient locomotion further.

In our model, the CoT had contributions from three elements, namely muscle synergy-based pulses and posture and speed regulators (ε ≃ ε^Syn^ + ε^height^ + ε^speed^). The muscle synergy-based pulses had the largest contribution and determined the basic U-shaped characteristics (Figure 10D). Although the posture and speed regulators had small contributions (Figure 10E), they had specific characteristics. In particular, while the posture regulator for walking remains almost constant with speed, that for trotting decreased, which moved the speed for the minimum CoT to the right (with respect to that for the muscle synergy-based pulses) and increased the difference in speed for the minimum CoT between walking and trotting. This allowed the model to achieve energy-efficient locomotion in a wider speed range. In contrast, the speed regulator increased with speed in both walking and trotting and had a similar shape against speed for walking and trotting, which had a small contribution to the difference in speed for the minimum CoT.

### 4.2. Gait Generation Based on Muscle Synergy

A large portion of motor commands in our model was generated by a linear combination of four rectangular pulses for each limb, where we used the same onset and end phases for the pulses between the four limbs. We changed the relative phase of the pulses between the forelimb and hindlimb to make the gait generation simple. However, a muscle synergy analysis of dogs showed that although a large portion of the muscle activity can be reproduced by a linear combination of four basic patterns for both forelimbs and hindlimbs in walking and trotting, as done for our model, the basic patterns had some differences, especially in the activation timings between forelimbs and hindlimbs and between walking and trotting (Deban et al., 2012; Catavitello et al., 2015). In particular, the basic patterns for the late extension and early and late flexion of the hindlimbs were earlier than those of the forelimbs in walking. The basic pattern for the early flexion of the hindlimbs was earlier than that of the forelimbs in trotting. The control of the activation timings of the muscle synergy patterns could contribute to the gait change (Cappellini et al., 2006; Aoi et al., 2019). In future studies, we would like to measure the trotting of rats and incorporate motor control differences between forelimbs and hindlimbs and between walking and trotting to clarify the gait-generation mechanism further.

### 4.3. Limitations of Our Model and Future Work

Although our simulation results showed features similar to those of animals (Figure 7), our model has limitations that prevent it from accurately reproducing animal locomotion. In particular, we did not incorporate the hand, phalangeal part of the hindlimbs, or flexibility of the spine in the trunk (Schilling and Hackert, 2006). These elements might improve the gait speed and energy efficiency. Furthermore, we confined our musculoskeletal model to two dimensions, which neglected instability in the lateral direction. More contribution of the posture regulator would be required for a three-dimensional model to maintain a stable posture during locomotion. In addition, although head movements are important and specific for gait (Zsoldos et al., 2010), we did not incorporate the neck. We would like to incorporate these features to clarify adaptive motor control mechanisms in animal locomotion further.

Not only the metabolic cost of locomotion but also other factors, such as musculoskeletal forces (Farley and Taylor, 1991), gait stability (Schöner et al., 1990; Diedrich and Warren, 1995; Aoi et al., 2013b), terrain and ground surface conditions (Prost and Sussman, 1969; Gustås et al., 2006; Goldenberg et al., 2008; Chateau et al., 2013), and genetic mutation (Andersson et al., 2012), influence the gait decision of animals. Furthermore, although animals change their gait smoothly when triggered by these factors, the gait transition mechanism also remains unclear. Our neuromusculoskeletal model will be useful for investigating these mechanisms in the future.

## Data Availability Statement

The datasets generated for this study are available on request to the corresponding author.

## Ethics Statement

The animal study was reviewed and approved by the Ethical Committee for Animal Experiments at the University of Tokyo.

## Author Contributions

SA developed the study design. MT performed the computer simulations and analyzed the data in consultation with SA, SF, TF, KT, and DY. MT and SA wrote the manuscript, and all the authors reviewed and approved it.

### Conflict of Interest

The authors declare that the research was conducted in the absence of any commercial or financial relationships that could be construed as a potential conflict of interest.

## Supplementary Material

Supplementary Movie S1 Simulated walking.

Supplementary Movie S2 Simulated trotting.
